# Low infectivity among asymptomatic patients with a positive SARS-CoV-2 admission test at a tertiary-care center, 2020–2022

**DOI:** 10.1017/ash.2023.218

**Published:** 2023-09-29

**Authors:** Ralph Tayyar, Melanie Kiener, Jane W. Liang, Gustavo Contreras Anez, Guillermo Rodriguez Nava, Alex Zimmet, Caitlin A. Contag, Krithika Srinivasan, Lucy Tompkins, Aruna Subramanian, John Shepard, Benjamin A. Pinsky, Jorge Salinas

## Abstract

**Background:** Many hospitals have implemented admission SARS-CoV-2 testing to evaluate for the need for transmission-based precautions. However, a positive test in an asymptomatic patient may represent (1) active infection, signifying infectiousness; (2) false positivity; or (3) past infection with prolonged viral shedding. We used a strand-specific SARS-CoV-2 reverse real-time polymerase chain reaction (rRT-PCR) assay to assess infectivity among asymptomatic patients with a positive SARS-CoV-2 PCR admission test. **Methods:** We used a 2-step rRT-PCR specific to the minus strand of the SARS-CoV-2 envelope gene. We reviewed records of patients with a positive SARS-CoV-2 PCR who were also tested for the strand-specific SARS-CoV-2 PCR within 2 days of admission at Stanford Health Care during July 2020–April 2022. We restricted our analysis to each patient’s first test. We calculated the percentage of detectable minus strand-specific tests among asymptomatic patients over time and gathered descriptive statistics for age, sex, and immunocompromised state. **Results:** In total, 848 admitted patients had strand-specific SARS-CoV-2 assays performed. Of 532 patients with a strand-specific assay done within 2 days of admission, 242 (45%) were asymp tomatic. Among asymptomatic patients, the mean age was 56 years (range, 19–99), 133 (55%) were male, 50 (21%) had immunocompromising conditions, and 30 (12%) were admitted for a surgical procedure. In total, 21 (9%; range, 4%–25% per quarter) had detectable minus strand-specific assays (Fig. 1). **Conclusions:** Most asymptomatic patients tested for SARS-CoV-2 on admission were not infectious. Hospitals using SARS-CoV-2 PCR admission testing may need to re-evaluate the continued use of this practice.

**Figure:**
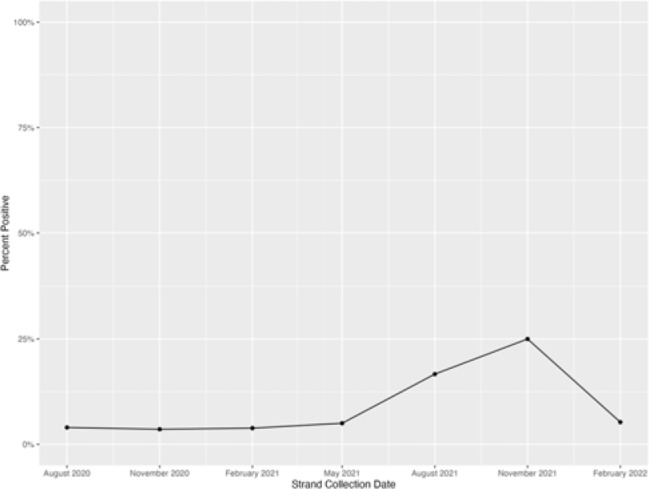

**Fig. 1.** Minus strand-specific SARS-CoV-2 assay percentage positivity per quarter among asymptomatic patients tested within 2 days of admission. The peak positivity in November 2021–January 2022 quarter coincided with the SARS-CoV-2 omicron variant surge in our county.

**Disclosure:** None

